# Posttraumatic Growth and Posttraumatic Depreciation: Independent Correlates of Well-Being Among People Living with HIV

**DOI:** 10.1007/s12529-022-10093-7

**Published:** 2022-04-29

**Authors:** Małgorzata Pięta, Marcin Rzeszutek

**Affiliations:** ªFaculty of Psychology, University of Warsaw, Stawki 5/7, 00-183, Warsaw, Poland

**Keywords:** Posttraumatic growth, Posttraumatic depreciation, Conservation of resources theory, Well-being, HIV/AIDS

## Abstract

**Background:**

Although posttraumatic growth (PTG) is intuitively associated with positive adaptation to traumatic life circumstances, studies regarding the link between PTG and well-being present mixed findings. Our study aimed to analyze the link between PTG and well-being indicators (resource gain and loss, positive and negative affect; PA/NA) in a clinical sample of people living with HIV (PLWH), with the additional control of parallel negative changes among participants, i.e., posttraumatic depreciation (PTD).

**Methods:**

The study sample comprised 509 PLWH who completed the standardized psychometric inventories measuring the study variables—PTG/PTD, resource gain and resource loss, and affective well-being.

**Results:**

By applying the person-centered perspective to the study results, we observed distinct clusters of participants within resources and PA/NA, which were uniquely associated with PTG/PTD levels, after controlling for sociomedical data among participants.

**Conclusions:**

Including two parallel sides of growth, i.e., PTG and PTD, our study may deepen the understanding of PTG mechanisms and processes among PLWH and inspire planning for more effective psychological interventions designed to meet the specific needs of these patients.

## Introduction

Twenty-five years have passed since Tedeschi and Calhoun [1, 2] introduced the concept of posttraumatic growth (PTG), which has become one of the leading research topics in positive psychology [3] and inspired other authors to create new models of psychological growth after trauma [e.g., 4–7]. Although empirical studies on PTG are extensive [see reviews and meta-analyses: 8, 9], several theoretical and methodological controversies regarding PTG have not yet been resolved [10]. More specifically, the experience of PTG is intuitively associated with positive adaptation to traumatic life circumstances, but studies on the link between PTG and well-being present mixed findings, with null or even negative relationships found among trauma survivors [see reviews and meta-analyses: 8, 9]. This raises doubts about the clinical utility of PTG in psychological research [5].

An important methodological limitation of contemporary PTG studies is that PTG measures assess positive changes after trauma only [11, 12], increasing the positive response bias [13]. Baker et al. [11] were the first authors to try to overcome this shortcoming by controlling parallel negative outcomes in the same PTG domains, which stimulated research on posttraumatic depreciation (PTD). Although several authors have found that PTG and PTD may co-exist with each other as independent trauma outcomes [12, 14], empirical evidence regarding PTG/PTD’s relationship to posttrauma well-being indicators remains scarce and inconsistent [15], particularly in clinical settings [16, 17].

The possibility of parallel, positive, and negative outcomes in the midst of adverse life challenges is highlighted by the conservation of resources theory [COR; 18, 19]. More specifically, while resource gain can have a protective function in the stress process, resource loss is characterized as a major predictor of psychological distress and poor well-being in the process of coping with stress [20]. Although COR underlines the role of gain and loss of resources primarily in the wake of ordinary stressors, Hobfoll et al. [6] also focused on traumatic stress and challenged the concept of PTG as an unequivocally salutogenic posttrauma outcome [see: 6, 21]. The correspondence between the constructs (PTG/PTD vs. resource loss/gain) is a matter of ongoing scientific discussion touching upon the primarily objective or subjective nature of growth after trauma and its significance for well-being [e.g., 20, 21]. In addition to the two paradigms, the simultaneous occurrence of positive and negative processes in the sequalae of stress and coping can also be viewed in Frederickson [22] broaden-and-build theory of affect, in which positive affect (PA) and negative affect (NA) are conceptualized as independent predictors and outcomes of adaptation to life stressors. Examination of the mutual association between PTG/PTD, with parallel control of resource loss/gain and finally PA/NA, can shed some light on PTG processes, especially in the process of coping with trauma associated with chronic illness [see: 23, 24].

In our study, we chose to analyze PTG/PTD processes in the context of the psychological well-being of people living with an HIV (PLWH). HIV diagnosis and living with its consequences are commonly associated with the experience of psychological trauma, associated with symptoms of posttraumatic stress disorder [PTSD; see: 25, 26]. However, at the same time, struggling with HIV infection can also act as a potential trigger of PTG [e.g., 27–29]. As with other trauma-exposed populations, the results concerning PTG among PLWH are inconsistent and share the same theoretical and methodological limitations [30]. The most common is following the variable-centered approach, which overlooks the heterogeneity of participants within the studied variables. In our study, we utilized a person-centered design [31], which can offer more insight into the link between PTG/PTD and well-being in this clinical population, known for its heterogeneous patterns of psychological and medical outcomes [30]. Specifically, the person-centered design enables observation of subgroups of PLWH that can differ substantially in their psychological and medical functioning after the diagnosis. Living with HIV itself is a dynamic experience dependent on the cultural and social context. Patients can have fundamentally different experiences associated with not only their diagnosis, depending on the time of their diagnosis, but also their specific personal characteristics. Thus, following the variable-centered approach only, i.e., treating these patients as homogenous group only because they are HIV positive [see contemporary studies on PTG in PLWH; 31], may preclude capturing the unique patterns of their psychological functioning. It is important especially nowadays, when HIV/AIDS is treated not as fatal disease but a chronic and manageable somatic problem [31].

## Current Study

Taking the aforementioned research gaps into consideration, the aim of our study was to investigate the relationship between PTG and PTD, and its positive (see PA and resource growth) and negative mental health outcomes (NA and resource loss) among PLWH, following the person-centered approach [31]. Our study takes into account existing research on:

1. PTG and PTD as independent predictors of well-being and adjustment outcomes among various trauma survivors [e.g., 14];
2. Hobfoll’s theory on the relationship between resource gain and loss and well-being outcomes [19], including traumatic stress and PTG [e.g., 6]; and
3. Fredrickson [22] broaden-and-build theory, in which PA may enhance personal resources, well-being, and psychological growth, but is independent of NA.

With these in mind, we formulated the following hypotheses:

### Hypothesis 1

In the entire sample of PLWH, there are distinct clusters of participants within PA/NA and resources, which are differently associated with PTG/PTD after controlling for sociomedical data among participants.

### Hypotheses 1a

Subjects belonging to clusters characterized by resource gain, high PA, and low NA report higher levels of PTG.

### Hypotheses 1b

Subjects belonging to clusters characterized by resource loss, high NA, and low PA report higher levels of PTD.

## Methods

The sample consisted of 509 participants infected with HIV between 1981 and 2019, 441 males (86.6%) and 68 females (13.4%) aged 20–77 years (*M* = 39.99; *SD* = 10.41), recruited from patients at the Hospital for Infectious Diseases in Warsaw. Out of 509 participants, 275 (54.0%) were in a stable relationship, 279 (54.8%) had higher education, 175 (34.4%) had secondary education, 41 (8.1%) had a vocational occupation, and 14 (2.8%) had elementary education. Most participants (377; 74.1%) had full employment, 55 (10.8%) were unemployed and subsisted on an illness allowance, and 21 (4.1%) were retired. The majority of participants had homosexual orientation (328; 64.4%), 136 (26.7%) had heterosexual orientation, and 45 (8.8%) defined their sexual orientation as other. Eighty participants (15.7%) were diagnosed with AIDS, and 51 (10.0%) had detectable viremia. The most frequent method of infection was sexual intercourse with a man, indicated by 360 participants (70.7%). Other methods included sexual intercourse with a woman (57; 11.2%), drug injection (47; 9.2%), and blood products (7; 1.4%). The period of antiretroviral treatment (ART) ranged from 6 months to 33 years (*M* = 7.21; *SD* = 5.57). CD4 level was in the range from 42.00 to 1,265.00 (*M* = 606.41; *SD* = 214.79).

Participants completed the informed consent form and then a paper-and-pencil version of the psychometric questionnaires, including the sociomedical survey. Participation in the study was voluntary, and no remuneration was granted. The eligibility criteria encompassed being 18 years and over, a medical HIV infection diagnosis, and currently being on ART. The exclusion criteria were related to HIV-related cognitive disorders, as assessed by medical doctors working in the hospital where the study was organized. Our study was approved by the local ethics committee.

## Measures

Expanded Version of the PTG and PTD Inventory [PTGDI-X; 32]. To measure PTG and PTD intensity among the participants, the 50-item PTGDI-X questionnaire was used in its Polish adaptation by Taku et al. [32]. PTGDI-X consists of positively stated items evaluating domains of PTG (five subscales: relating to others, new possibilities, personal strength, spiritual change, and appreciation of life; e.g., *I am more willing to express my emotions*) along with the same items, but formulated negatively and measuring PTD [e.g., *I am less willing to express my emotions*; 33]. Responses are given on a 6-point scale, ranging from 0 (*I did not experience this change*) to 5 (*I experienced this change to a great degree*). Higher scores indicate more intense PTG or PTD levels. We followed the global PTG and PTD scores according to Taku et al. [32] study. Participants were instructed to focus on the positive or negative changes following their lives after receiving their HIV diagnosis The Cronbach’s alphas for the global PTG and PTD scores, as well as particular subscales in this tool, varied between 86 and 97.

Conservation of Resources Evaluation (COR-E; 18]. Resource gain and loss were assessed by the short version of the COR-E questionnaire, the Polish adaptation by Dudek et al. [34]. In the COR-E, there are 40 items describing resources related to family, power, vitality, wealth, and spirituality. Participants were asked to state the extent to which they had experienced gains or losses in each of the resource categories from 0 (*no change*) to 5 (*a very large loss/gain*). On this basis, after summing the results, two indicators were obtained: the indicator of resource gain and the indicator of resource loss. Higher values indicate higher losses or gains. We instructed our participants to focus on the potential gain or loss of resources in the aftermath of struggling with HIV infection. The Cronbach’s alphas for the COR-E scores were equal for both gain and loss scale, i.e., 0.97.

Positive and negative affect were evaluated with 20 descriptions of feelings and emotions: 10 for PA (e.g., “proud” and “excited”) and 10 for NA (e.g., “depressed” and “stressed”), taken from the PANAS-X by Watson et al. [35]. Participants assessed their general affective states on a 5-point response scale from 1 (*not at all*) to 5 (*strongly*). Cronbach’s alpha coefficients obtained in this study for PA and NA varied between 0.87 for PA and 0.91 for NA.

### Data Analysis

The data analysis in the current study was performed in three stages. First, descriptive statistics for the analyzed variables were calculated to assess the variable distributions. Second, to extract possible profiles of loss/gain levels with positive and negative affect, cluster analysis based on the k-means method was applied [36]. The k-means clustering method is used in non-hierarchical cluster analysis, which is being used for division of a set of objects into a predefined number (k) of clusters. The criterion for such subdivision in this study was the minimal dispersion inside clusters. The extracted clusters were evaluated also in terms of sociomedical data (see gender, age, relationship status, education, employment, financial situation, sexual orientation, substance use, mode of infection, HIV infection duration, period of ARV treatment, AIDS phase, and CD4 count). Third, to assess the relationships between profiles of loss/gain levels PTG/PTD levels, multivariate analysis of variance was performed. Because of the nonnormality of the analyzed variables, post hoc tests based on the bootstrap method were applied.

## Results

Table 1 presents the descriptive statistics for the analyzed variables, which were positive and negative affect, resource loss and gain, and PTG/PTD levels. The four clusters were distinguished based on the six research variables. Statistically significant relationships between the sociomedical variables and the extracted clusters were described at the last step of the analysis. The distribution of PTD and the level of loss were positively skewed and leptokurtic, while the distribution of PTG was platykurtic. The correlation between PTG and PTD in the current study was statistically significant and positive, but very weak, *r* (507) = 0.14, *p* < 0.01. However, the distributions of positive affect, negative affect, and the level of gain did not differ from the normal distribution in terms of skewness and kurtosis.

**Table 1:** Descriptive statistics and correlation matrix for analyzed variables

Variables	*M*	*SD*	*S*	*K*	2	3	4	5	6
1. Positive affect	33.19	7.31	− 0.46	− 0.04	− .44; − .29	− .34; − .17	.11; .28	.12; .29	− .38; − .22
2. Negative affect	21.37	8.65	0.85	0.08	-	.22; .38	− .22; − .04	− .10; .08	.29; .44
3. Loss	.62	0.88	1.93	3.34	-	-	− .20; .16	.03; .21	.45; .59
4. Gain	1.33	1.20	0.72	− 0.41	-	-	-	.55; .66	− .04; .14
5. PTG	52.37	33.97	.05	− 1.15	-	-	-	-	.05; .22
6. PTD	22.62	25.36	1.27	1.02	-	-	-	-	-

*M* Mean Value, *SD* Standard Deviation, *S *skewness, *K* kurtosis

The levels of loss and gain did not correlate with each other, *r* (507) = 0.07, *p* > 0.05. Positive affect and negative affect correlated negatively, *r* (507) =  − 0.37, *p* < 0.001.

**Fig. 1:**
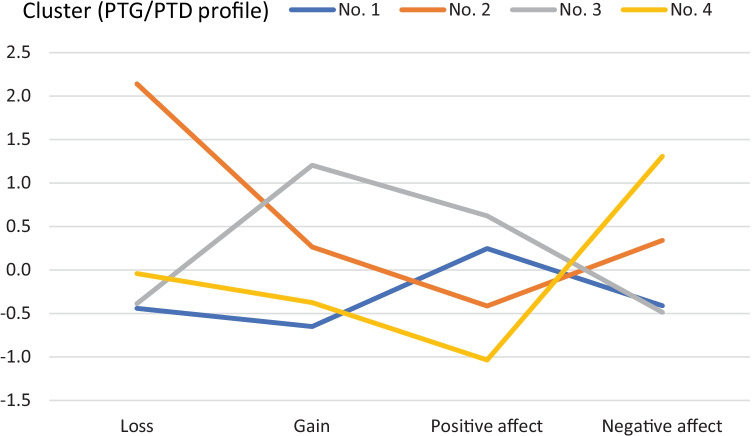
Profiles of PTG and PTD in extracted clusters

The level of gain, level of loss, positive affect, and negative affect were analyzed using cluster analysis based on the k-means method. Four distinct clusters were extracted. Figure 1 presents the standardized values of gain, loss, positive affect, and negative affect in the extracted clusters. The first cluster consisted of participants (*n* = 187), with average levels of gain and loss of resources and average intensity of PA/NA. The second cluster consisted of participants (*n* = 59) with a high level of loss, average level of resources gain, and average intensity of PA/NA. The third cluster comprised participants (*n* = 115) with high levels of gain and average levels of loss in resources, high levels of PA, and average NA. The final, fourth cluster consisted of participants (*n* = 84) with average levels of loss and gain, low levels of PA, and high levels of NA. Participants with missing data on level of gain, level of loss, positive affect, or negative affect were excluded from clustering.

**Fig. 2:**
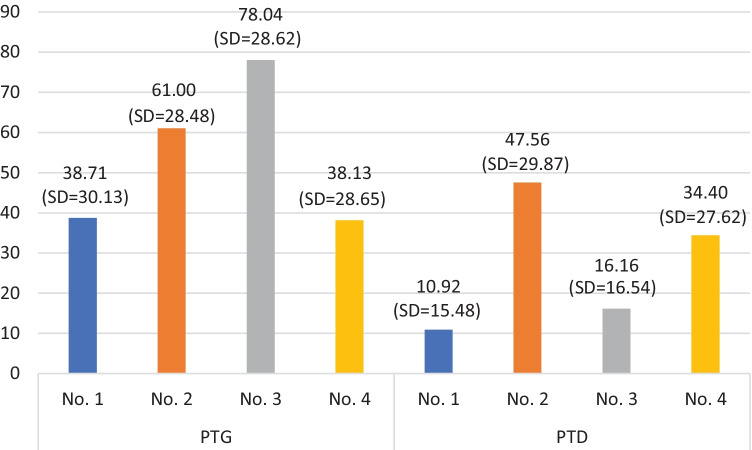
Mean values of PTG and PTD in extracted clusters

In the next step of the data analysis, the differences between the extracted clusters in terms of PTG and PTD levels were analyzed. Multivariate analysis of variance revealed that the between-group differences were statistically significant, *F*(2,432) = 831.83, *p* < 0.001, *η*^2^ = 0.79. Subsequent analysis of variance revealed that the extracted clusters differed both in terms of PTG levels, *F*(3,433) = 49.36, *p* < 0.001, *η*^2^ = 0.26, and PTD levels, *F*(3,433) = 61.15, *p* < 0.001, *η*^2^ = 0.30. According to the values of the Gabriel post hoc test based on the bootstrap method, cluster no. 1 differed significantly in PTG from clusters no. 2 [− 30.45; − 13.79] and no. 3 [− 45.67; − 32.62]. Also, cluster no. 4 differed significantly in PTG from clusters no. 2 [− 32.43; − 11.93] and no. 3 [− 47.61; − 31.46], and cluster no. 2 differed significantly from cluster no. 3 [− 26.80; − 8.37]. PTG was highest in cluster no. 3 (loss-avg., gain-h, PA-h, NA-avg.), lower in cluster no. 2 (loss-h, gain-avg., PA-avg., NA-avg.), and lowest in clusters no. 1 (loss-avg., gain-avg., PA-avg., NA-avg.) and no. 4 (loss-avg., gain-avg., PA-l, NA–h) (see Fig. 2).

According to the values of the Gabriel post hoc test based on the bootstrap method, cluster no. 1 differed significantly in terms of PTD from clusters no. 2 [− 45.91; − 29.85], no. 3 [− 8.94; − 1.15], and no. 4 [− 30.05; − 16.51]. Also, cluster no. 2 differed significantly in PTG from clusters no. 3 [24.59; 41.09] and no. 4 [4.35; 23.65], and cluster no. 3 differed significantly from cluster no. 4 [− 25.15; − 11.63]. PTD was highest in cluster no. 2 (loss-h, gain-avg., PA-avg., NA-avg.), lower in cluster no. 4 (loss-avg., gain-avg., PA-l, NA–h), even lower in cluster no. 3 (loss-avg., gain-h, PA-h, NA-avg.), and lowest in cluster no. 1 (loss-avg., gain-avg., PA-avg., NA-avg.) (see Fig. 2).

Table 2 presents the statistically significant relationships between the sociomedical variables and the extracted clusters.

**Table 2:** Statistically significant relationships between extracted clusters and sociomedical data

Variable	Cluster no. 1	Cluster no. 2	Cluster no. 3	Cluster no. 4	
	*N* (%)	*N* (%)	*N* (%)	*N* (%)	
Higher education	117 (62.6%)	26 (44.1%)	54 (47.0%)	50 (59.5%)	*χ*^2^(3) = 10.85, *p* < .05
Full employment	149 (79.7%)	28 (47.5%)	88 (76.5%)	62 (73.8%)	*χ*^2^(3) = 24.74, *p* < .001
Non-heteronormative orientation	157 (84.0%)	39 (66.1%)	70 (60.9%)	64 (76.2%)	*χ*^2^(3) = 22.15, *p* < .001
HIV diagnosis/period of ARV treatment (*M* ± *SD*)	6.39 ± 4.98	8.85 ± 6.02	8.25 ± 5.89	6.76 ± 5.74	*F*(3,441) = 4.65, *p* < .01

*M* Mean Value, *SD* Standard Deviation, *χ*^*2*^ Chi-squared test for independence, *df*, Degrees of freedom, *p* statistical significance, *F* one-way analysis of variance

As can be seen from the controlled sociomedical data, higher education, employment, non-heteronormative sexual orientation, and time since diagnosis/treatment were significantly correlated with belongingness to the extracted clusters.

## Discussion

Our results were mainly in accordance with our research hypotheses, as we observed four distinct clusters of study participants that differed in the context of resources and affect, which we also related differently to the PTG/PTD intensity among participants. More specifically, the first cluster, which was the largest, was characterized by average levels of both resource loss and gain, and average intensity of PA and NA. Participants from this cluster also experienced low levels of both PTG and PTD. It seems that subjects belonging to this cluster represented relative adaptation to living with HIV with a balance of loss and gain experiences [30], protecting them from entering the loss spiral described by Hobfoll [18, 19]. This phenomenon may be due to, e.g., stable personality characteristics or specific cultural context, which should be examined in the future studies [37].

In contrast, the participants from the second cluster reported a high level of resource loss, accompanied by average levels of all other controlled dimensions. These subjects experienced high PTD levels, which is consistent with the foundation of the COR theory between objective resource losses and depreciation of different areas of life [6], empirically tested in the case of PTG with chronic illness [24]. Further, the third cluster of participants was characterized by high resource gains with high levels of PA, and the lowest PTD. In other words, the third cluster comprised the group of PLWH with the highest perceived benefits following HIV infection that were also reflected in their well-being [30]. Consequently, these groups seem to reflect objective benefits and losses, which are reflected in employment status or level of education, that can be linked to experiencing PTG and PTD phenomena.

Finally, PLWH from the fourth cluster reported average levels of resource loss and gain, low levels of PA and high levels of NA, and low PTG along with average PTD. This last cluster of patients was also characterized by poor affective well-being. This finding suggests a significant association between affective well-being and PTG and is indirectly consistent with Frederickson’s model [22], where low PA and high NA can undermine cognitive processes that lead to redefinition of the current situation and thus PTG [1, 2]. As this group was on average most recently diagnosed with HIV, this condition may be linked to nonsufficient time for PTG processes to occur.

It should be noted that distinct clusters of participants varied in their sociomedical variables in our sample. From the sociodemographic data, higher education, full employment, and non-heteronormative sexual orientation were significant correlates of belongingness to more “adaptive” clusters (i.e., generally with relatively high resource gain, high well-being, and high PTG and low PTD), which is consistent with some reviews on that topic [30]. We did not observe significant association between the rest of examined sociodemographic variables, such as gender, age, relationship status, or financial situation with specific clusters. It is worth noting that our sample can be described as highly homogenous in terms of sociodemographic characteristics. Moreover, all participants were of Polish nationality and Caucasian ethnic background. Of the medical variables, only the time since HIV diagnosis and years of treatment were associated with resources and well-being clusters. Interestingly, we did not observe significant differences in rates of participants that entered AIDS stage across the clusters. Moreover, only the time that elapsed since diagnosis, independently from patient’s age, was a predictor of cluster participation. Participants from two contrasting clusters, i.e., the second (highest PTD) and third (highest PTG), were those with the longest time elapsed since their HIV diagnosis, while participation in the first and fourth clusters was associated with the shortest time spent living with HIV. This finding is consistent with Tedeschi and Callhoun’s PTG model and may reflect the time necessary for PTG processes to develop [1, 2]. It also seems that time is also needed for PTD to appear, yet longitudinal studies are required to confirm this hypothesis. Longitudinal study design can also address the causes of higher general levels of PTG among the participants compared to the PTD scores. Finally, our findings may reflect a process of illness adaptation and return to the baseline level of affective well-being after a long life with HIV [38].

### Strengths and Limitations

Our study has several strengths, including analysis of both sides of growth, i.e., PTG and PTD in parallel in a large clinical sample of PLWH, additionally employing the person-centered perspective. Nevertheless, this research is not free of limitations. First, due to the cross-sectional design of the study, no cause-and-effect relationships between the measured variables can be drawn from the results. Second, the study sample was heterogeneous regarding sociomedical variables, with time since HIV diagnosis and age in particular. In the future studies, it would be also very interesting to disentangle the effects of HIV-related clinical variables from the chronological age of participants. Further, although we implemented parallel PTG and PTD measurements accompanied by resource gains and losses and binary affective well-being indicators, all measures in our study were subjective and retrospective. Future studies should focus on the daily representations of PTG/PTD in everyday life after trauma [10].

## Conclusions

Despite its apparent limitations, the study provides a new understanding of PTG/PTD processes among people living with HIV. More specifically, the mutual association between PTG and well-being among trauma survivors remains a research gap, full of studies with inconsistent results [see reviews and meta-analyses; 4, 8, 9]. Our study offered insight into this topic from a different angle. It seems that personal resources may translate into both sides of growth, i.e., PTG and PTD, which can be seen as independent correlates of PLWH’s well-being. The key implication of this finding may be an incentive to monitor the PTG and PTD processes in clinical work with patients living with HIV as they can reflect objective gains and losses in psycho-social and material resources linked to living with HIV. This topic should be explored through further research, as it may offer insight into the debate on the status of PTG through the lens of COR theory [6, 21]. Finally, one should remember that different patterns of growth and depreciation can be observed in coping with HIV/AIDS, which should be taken into an account in psychological counselling among patients living with HIV.

## Declarations

### Research Involving Human and Animal Participants

All procedures performed in studies involving human participants were in accordance with the ethical standards of the institutional and/or national research committee and with the 1964 Helsinki Declaration and its later amendments or comparable ethical standards.
